# Physical-chemical gradients, CO_2_ venting dynamics and microbial community composition in a shallow Mediterranean CO_2_-rich hydrothermal system

**DOI:** 10.1038/s41598-026-57265-z

**Published:** 2026-06-13

**Authors:** Juan Pablo Martín-Díaz, Alba González-Vega, Clàudia Pérez-Barrancos, Jesús M. Arrieta, Desirée Palomino, Ignacio Baena-Vega, Sandra Mallol, Nuria R. de la Ballina, Irene Díez, Juan T. Vázquez, David Díaz, Eugenio Fraile-Nuez

**Affiliations:** 1 Centro Oceanográfico de Canarias, Instituto Español de Oceanografía, Consejo Superior de Investigaciones Científicas (IEO-CSIC), 38180 Santa Cruz de Tenerife, Spain; 2 Universidad de La Laguna (ULL), San Cristóbal de La Laguna, 38200 Spain; 3 Centro Oceanográfico de Málaga, Instituto Español de Oceanografía, Consejo Superior de Investigaciones Científicas (IEO-CSIC), Málaga, 29002 Spain; 4 Centro Oceanográfico de Baleares, Instituto Español de Oceanografía, Consejo Superior de Investigaciones Científicas (IEO-CSIC), 07015 Palma de Mallorca, Spain; 5 Servicios Centrales, Instituto Español de Oceanografía, Consejo Superior de Investigaciones Científicas (IEO-CSIC), 28002 Madrid, Spain

**Keywords:** Shallow hydrothermal system, Calent mound, CO_2_ emissions, PH anomaly, Nutrient enrichment, Microbial community., Biogeochemistry, Climate sciences, Ecology, Ecology, Environmental sciences, Ocean sciences

## Abstract

Shallow-water CO_2_-rich hydrothermal systems provide natural laboratories for studying localized ocean acidification under realistic environmental conditions. Here, we present a multidisciplinary characterization of the Calent mound CO_2_-rich system (Columbretes Islands, Western Mediterranean), based on oceanographic surveys conducted in 2020 and 2021. Localized pH anomalies were detected directly above active vents, reaching maximum reductions of 1.12 pH units, whereas water-column temperature anomalies were minimal and subsurface sediment temperatures exceeded ambient seawater by 5.67 °C. Gas analyses indicated high CO_2_ concentrations (0.094 ± 0.008 mol L^− 1^), with heterogeneous degassing regimes, ranging from sporadic to continuous emissions and an average flux of 189.4 ± 15.4 kg CO_2_ m^− 2^ yr^− 1^ at the active vent field. Vent fluids were significantly enriched in dissolved inorganic nutrients, particularly silicate, phosphate, nitrate+nitrite, and ammonium. Benthic microbial mats hosted metabolically diverse prokaryotic and eukaryotic communities, including hydrothermal-associated taxa such as Zetaproteobacteria, Campylobacterota, and Nitrosophaeria, consistent with iron, sulfur, and ammonia oxididation metabolisms. Several microbial core taxa persisted across years despite shifts in relative abundance. These findings demonstrate that Calent mound sustains an intense yet highly localized biogeochemical environment within the photic zone, where CO_2_ venting and nutrient inputs jointly influence carbonate chemistry and microbial community structure.

**Supplementary Information:**

The online version contains supplementary material available at https://doi.org/10.1038/s41598-026-57265-z.

## Introduction

Oceans play a vital role in regulating global processes, including the absorption of atmospheric CO_2_. It is estimated that since 1850, the oceans have removed 26% of total anthropogenic emissions^1^. As a consequence, since the pre-industrial era, average surface ocean pH has declined by approximately 0.1 units, with further reductions projected under continued CO_2_ emissions reaching up to 0.5 pH units by 2100^2,3^.

Despite natural CO_2_-rich hydrothermal systems contributing to the global carbon budget, their impact is estimated to be less than 0.5% of anthropogenic CO_2_ emissions^4^. These systems can significantly alter local ocean chemistry and generate steep chemical gradients over small spatial scales, providing a realistic framework to evaluate ecological responses to sustained acidification^5^.

Shallow-water hydrothermal vents (< 200 m depth) are particularly valuable natural laboratories because their influence occurs within the photic zone, where CO_2_ venting directly interacts with primary production and complex food webs. Detailed characterization of such systems is therefore essential to validate long-term projections derived from experimental and modelling approaches^4^, and to assess their broader biogeochemical and ecological significance under realistic field conditions. In the Mediterranean Sea, several shallow hydrothermal sites have been described across a range of geological and oceanographic settings^6–9^, yet many remain insufficiently characterized in terms of CO_2_ flux magnitude, spatial extent of acidification, and associated biological composition. Among these, the Calent mound hydrothermal system in the Columbretes Islands (Western Mediterranean) represents a particularly valuable study site. Its accessible depth enables direct fluid and biological sampling by scuba divers, while the steep pH gradients it generates, encompass acidification scenarios projected under high-emission trajectories for 2050–2100^2,3^. Furthermore, Calent mound lies within the Columbretes Islands Marine Reserve, designated as a Natura 2000 site under a Site of Community Importance (SCIs ESZZ16010) pursuant to the EU Habitats Directive (92/43/CEE). Notably, the active venting system falls within Habitat 1180 (“Submarine structures made by leaking gases”), a habitat of Community interest explicitly listed in Annex I of the Directive, underscoring the conservation relevance of characterizing this system. This protected status provides a rare opportunity to study hydrothermal dynamics largely free from direct anthropogenic stressors such as fishing or coastal pollution. Despite previous observations reporting elevated pCO_2_ and shifts in benthic community composition^7^, the spatial distribution of hydrothermal anomalies, the magnitude and temporal variability of CO_2_ emissions, associated nutrient inputs, and the composition of microbial communities remain insufficiently characterized, limiting our understanding of its biogeochemical and ecological significance.

In this study, our goal was to characterize physical and chemical anomalies associated with venting activity, and evaluate the likely impact on microbial community structure, linking hydrographic and microbial community observations to assess how geochemical gradients shape community composition. Together, these observations provide a comprehensive description of a shallow CO_2_-rich hydrothermal system in the western Mediterranean and establish a baseline for evaluating its environmental variability and ecological significance.

## Results

**Fig. 1:**
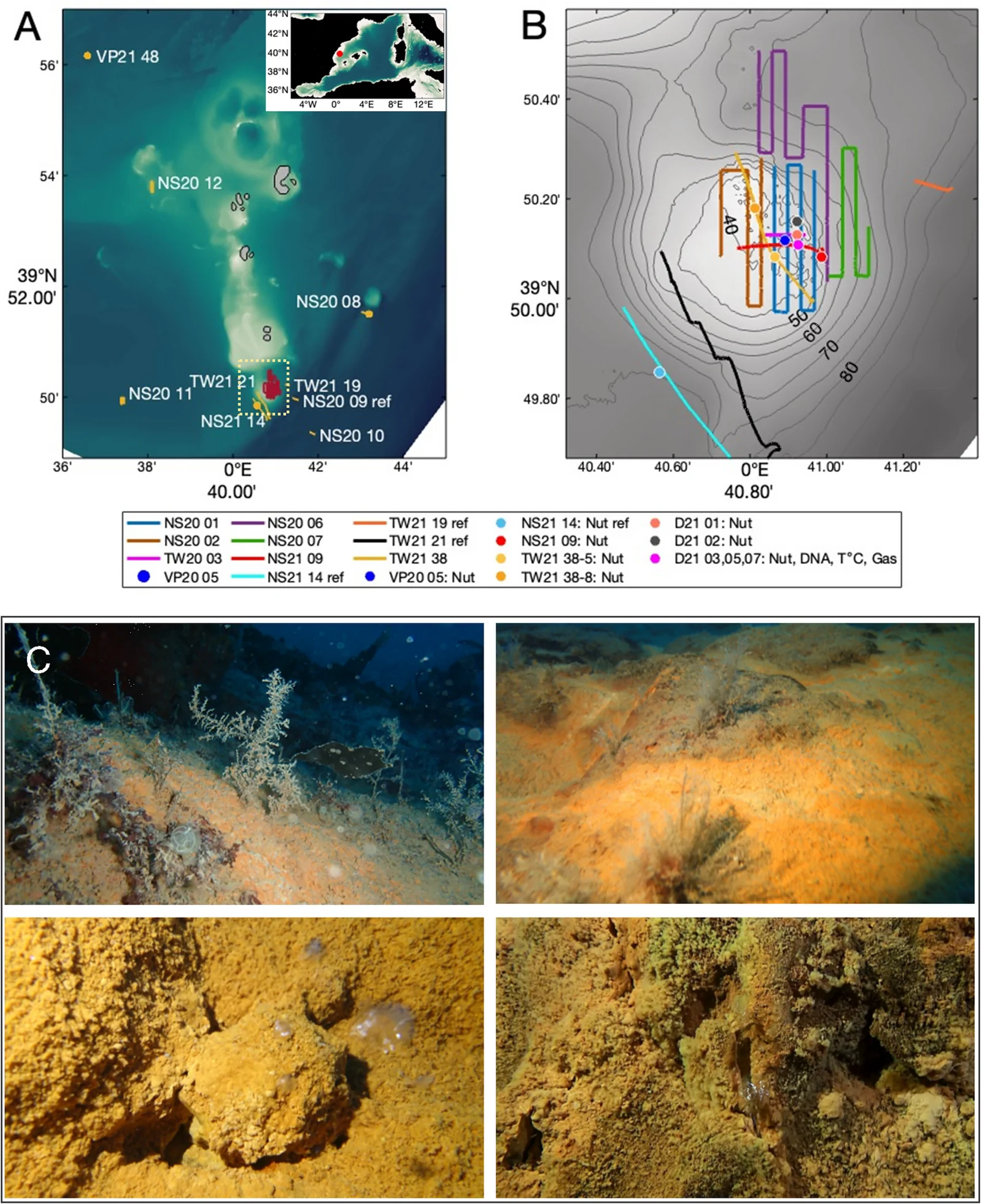
Map showing the study area. (**A**) Location of the Columbretes Islands in the Mediterranean Sea (red dot in embedded map), reference stations (yellow transects for hydrographic references and yellow dots for nutrient references) and location of Calent mound in the south of the archipelago (yellow dashed-line square), where the study is mainly carried out (red transects); (**B**) hydrographic survey and water sampling in Calent mound, where NS – near seabed tow, VP – vertical profile, TW – tow-yo, D – Dives, Nut – Nutrients samples, DNA – microbial composition study, Gas – gas samples and gas bubbling video recording. Numbers after sampling technique indicate cruise year (20 for 2020 and 21 for 2021). (**C**) Imagery from the hydrothermal active area, where microbial mats cover actively bubbling vents. The map in panel A was generated using MATLAB R2021a (The MathWorks Inc., Natick, MA, USA; https://www.mathworks.com/products/matlab.html).

We are reporting results based on two multidisciplinary oceanographic surveys conducted in 2020 (Colcarto-0220) and 2021 (Colcarto-0221) at the Calent mound hydrothermal system (Fig. 1). This dataset combines high-resolution hydrographic measurements with direct gas and fluid sampling. In addition, we quantify CO_2_ emission fluxes, assess dissolved inorganic nutrient enrichment, and describe the composition of prokaryotic and eukaryotic microbial communities inhabiting hydrothermally influenced areas.

### Hydrographic variability

**Fig. 2:**
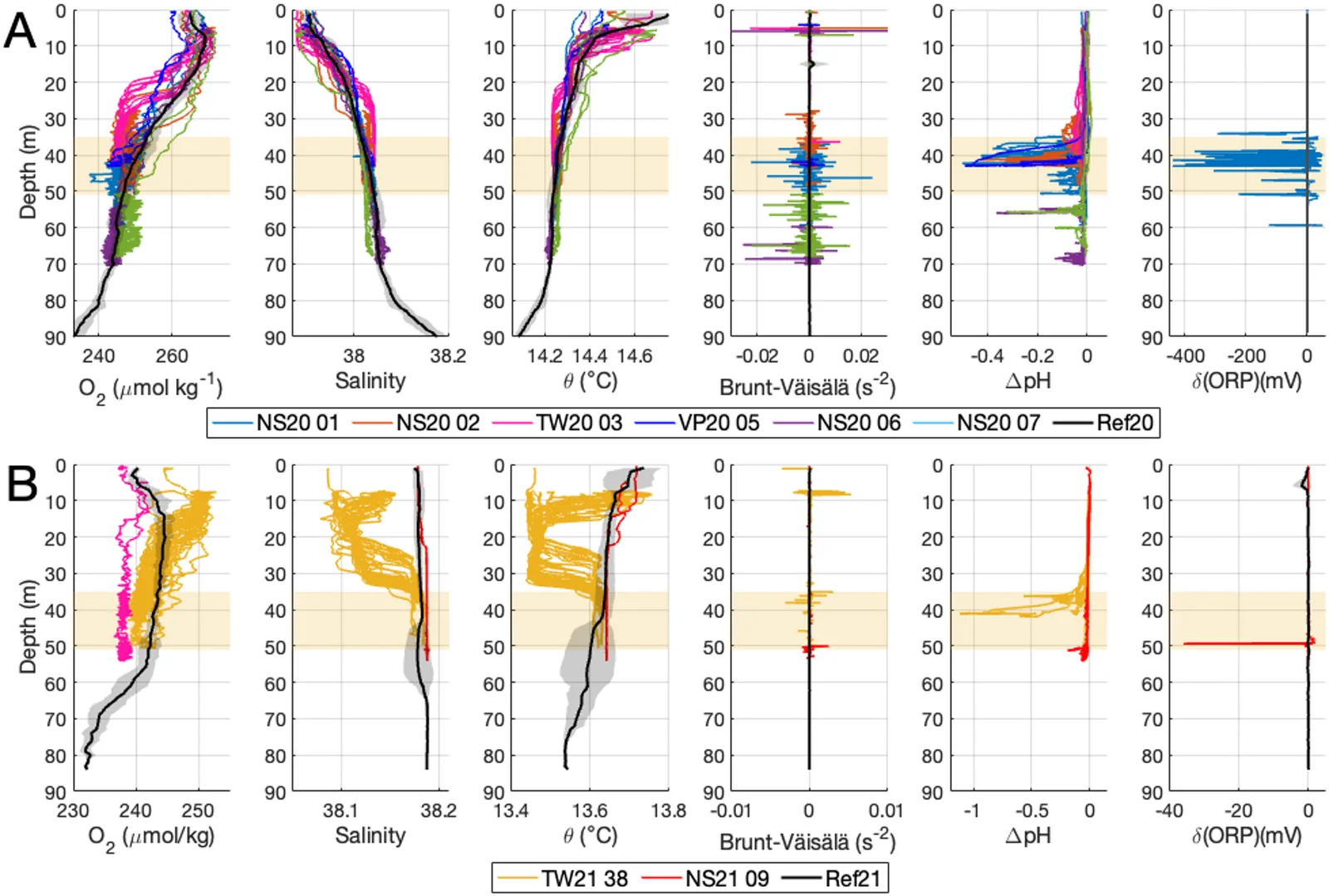
Vertical profiles of the physical-chemical properties of the seawater column. (**A**) Profiles from the 2020 cruise and (**B**) profiles from the 2021 cruise. Yellow bands indicate the depth range of the main active venting area (35–51 m). Profiles below the yellow band correspond to tow-yo measurements acquired over the flanks of the mound. Grey shaded areas indicate the standard deviation of average values of reference stations (black lines). ΔpH represents the pH anomaly relative to reference stations, and δORP represents the rate of change of ORP between consecutive measurements.

The water column above the active field revealed relatively stable physical-chemical conditions in 2020 (Fig. 2, top), while substantial spatial and temporal variability was observed in the 2021 survey (Fig. 2, bottom), particularly between stations NS21 09 and TW21 38, surveyed just 5 days apart. At vent-influence depths (approx. 35–51 m depths), hydrographic profiles revealed localized anomalies in dissolved oxygen, salinity and temperature relative to reference waters. At vent depths, minimum dissolved oxygen concentrations reached 238 µmol kg^− 1^ in 2020 and 236 µmol kg^− 1^ in 2021, showing decreases of 11 µmol kg^− 1^ and 6 µmol kg^− 1^, respectively, compared to reference waters. Salinity and temperature displayed minor fluctuations (± 0.02 and ± 0.05 °C, respectively) compared to reference profiles, however, certain instability was observed at the depth of the hydrothermal system, as indicated by Brunt-Väisälä frequency calculations. Subsurface temperature measurements (20 cm into the sediment) revealed average temperature of 18.21 ± 0.60 °C, with maximum values of 19.08 °C, decreasing rapidly toward sediment surface with 15.30 ± 1.49 °C and overlying water (20 cm above seabed), where temperature reached ambient seawater temperature with 13.52 ± 0.03 °C. Maximum temperature differences of up to 5.67 °C were recorded across the 40 cm vertical gradient during the four-day deployment period.

### pH and ORP anomalies

Distinct negative pH anomalies were detected above the hydrothermal field during both surveys (Fig. 2). Maximum pH decreases reached 0.50 units in 2020 and 1.12 units in 2021, centered at depths of 40–45 m. Redox potential anomalies (δORP) co-occurred with pH reduction at venting depths during both surveys. An exception is TW21 38 in 2021, where ORP data were unavailable due to sensor malfunction, while pH data for this station were recorded correctly. No notable pH or ORP anomalies were detected in surface waters, indicating that acidification effects were restricted to the vicinity of the vent field.

**Fig. 3:**
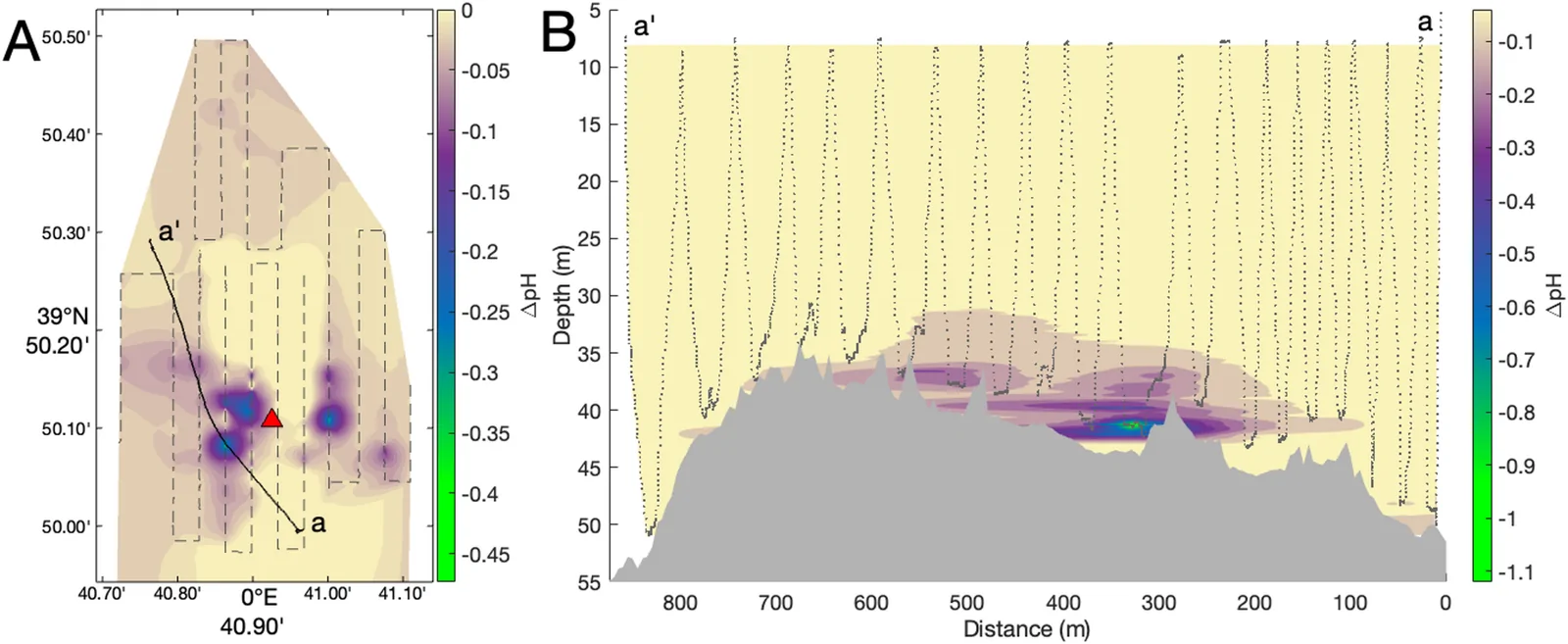
Spatial distribution of the pH anomalies. (**A**) Horizontal contour from 2020 survey, where tracks of NS tows represented by grey dashed lines, and location of video recording marked with a red triangle. (**B**) Vertical contour from 2021 survey (track of TW represented by grey dots) through transect a-a’ previously indicated in (A). Color scales differ between panels A and B to accommodate the distinct range of pH anomalies observed during each survey year.

Spatial mapping revealed heterogeneous distribution of pH anomalies (Fig. 3). In 2020, Calent mound was explored by NS tows technique (see Methods section), identifying a − 0.05 pH anomaly zone extending ~ 70,460 m², while areas exposed to anomalies of − 0.10 pH units covered ~ 17,200 m² (equivalent to the 41% and 10%, respectively, of the total mapped mound area of 171,500 m^2^), mainly located in the NE sector of the mound. In 2021, TW surveys showed hydrothermal plumes with negative anomalies greater than − 0.05 pH units mainly restricted up to 10–15 m above the seafloor, with a cross-sectional plume area of 2,600 m^2^ along the TW21 38 transect, and with intensity increasing closer to the seabed.

### CO_2_ bubbling dynamics and fluxes

**Fig. 4:**
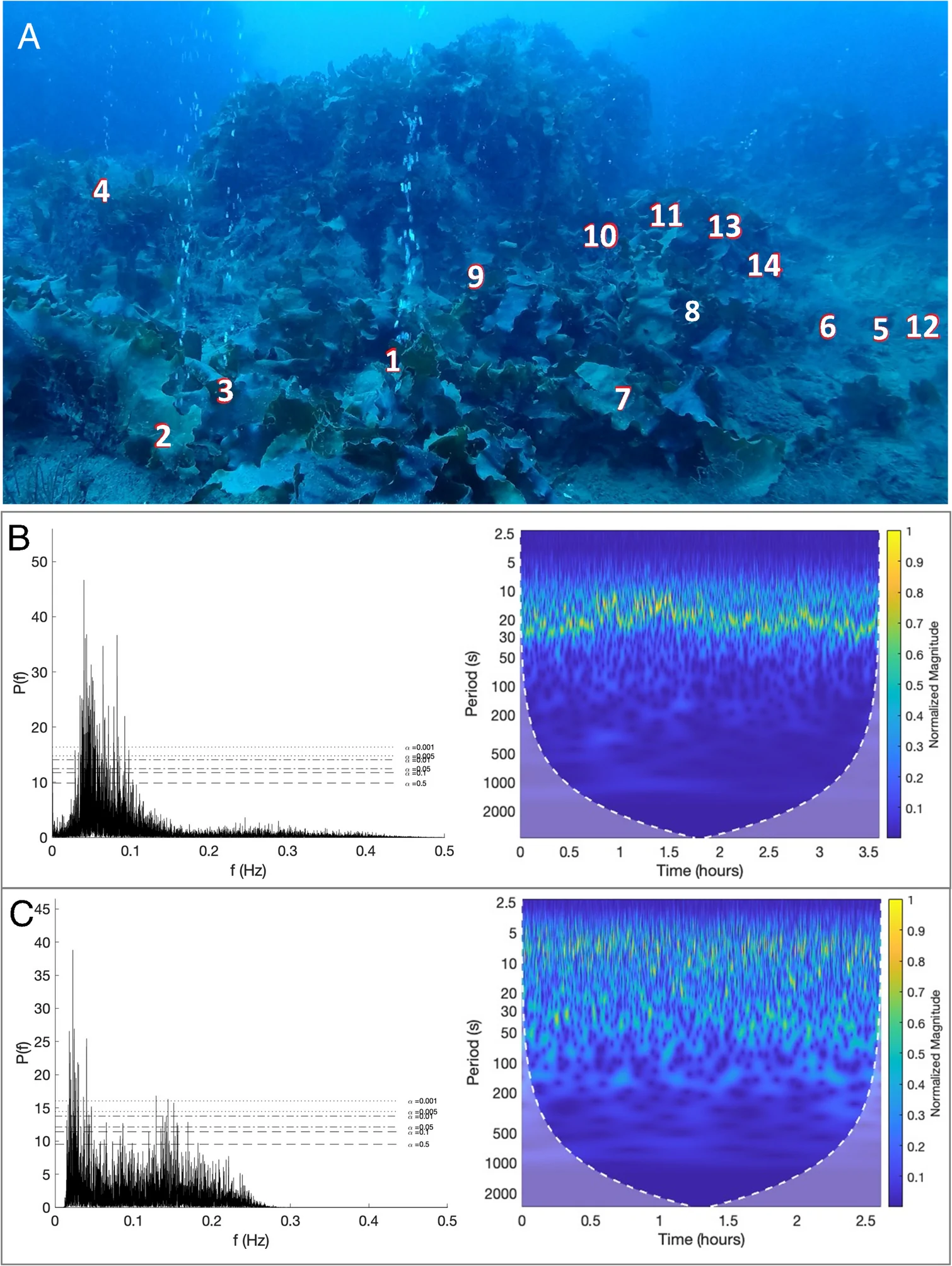
Study of periodicity on the gas emissions. (**A**) Footage of the video with the study area containing the 14 bubbling vents; (**B**) FFT (left) and Wavelet (right) analysis of the vent 3; (**C**) FFT (left) and Wavelet (right) analysis of the vent 14.

Two complementary techniques to study periodicities in the gas emissions were applied: Fast Fourier Transform (FFT), which decomposes a time series into its constituent frequencies to identify dominant emission periodicities, and Wavelet Transform (WT), which also identifies periodicities and additionally provides their temporal location within the time series, making it particularly suited for non-stationary signals as those with abrupt changes or intermittent cycles. This analysis was performed on 11 vents to assess temporal emission dynamics, excluding the 3 vents (ID 4, 10 and 11) that exhibited continuous degassing throughout the recording period and for which periodicity analysis was not applicable. The remaining vents displayed distinct emission cycles as observed in Supplementary Table S1. FFT analysis identified some vents emitting at only one range of significant frequency, while others displayed multiple significant cycles, with short- and long-period emissions (e.g., Fig. 4). WT analysis aligned with the FFT findings, highlighting temporally localized cycles along the time dimension.

The average gas outflow rate was 6.79 ± 0.56 L h^− 1^ and average CO_2_ concentration of 0.094 ± 0.008 mol L^− 1^ in the gas-phase. Fluxes from individual vents showing periodicity ranged from 7.18 ± 0.59 to 226.31 ± 18.43 kg CO_2_ yr^− 1^, while estimations for vents with continuous degassing averaged 243.57 ± 19.84 kg CO_2_ yr^− 1^ (Supplementary Table S1). In Table 1, vents were sorted based on the duration of active bubbling, as continuously active (> 50 min h^− 1^), regularly active (10–50 min h^− 1^), or sporadically active (< 10 min h^− 1^). Although continuously active vents represented ~ 42% of observed vents, they accounted for nearly 74% of the total CO_2_ output within the surveyed ~ 10 m^2^ area (Fig. 4A). The estimated average CO_2_ flux in this area was 189.4 ± 15.4 kg CO_2_ m^− 2^ yr^− 1^. Spatially scaled annual CO_2_ emission estimates derived from this flux are summarized in Table 1, using four reference areas of increasing spatial extent: two based on the spatial footprint of pH anomalies (≥ −0.2 pH units; ~ 3,100 m^2^; and ≥ −0.1 pH units, ~ 17,200 m^2^), and two based on the officially mapped extent of Habitat 1180 (“Submarine structures made by leaking gases”, Annex I, EU Habitats Directive 92/43/CEE) within the Columbretes Islands Natura 2000 network (SCI ESZZ16010). The Standard Data Form for SCI ESZZ16010 registered 6.86 ha^10^, while the updated mapping conducted by the INTEMARES project reported a substantially larger extent of 164 ha across Columbretes Islands, including hydrothermally active areas beyond Calent mound^11^.

**Table 1:** CO_2_ flux regimes in the study area of ~ 10 m^2^. Average and total fluxes are calculated per flux regime. Spatially scaled annual CO_2_ emission estimates derived from four reference areas of increasing spatial extent. Flux estimates are based on an average flux of 189.4 ± 15.4 kg CO_2_ m^− 2^ yr^− 1^ measured at the active vent field.

Flux category	ID vents	Average time of emission (min) in 1 h	Average CO_2_ flux/vent (kg CO_2_ yr^− 1^)	Total CO_2_ flux in the study area (kg CO_2_ yr^− 1^)		Contribution to CO_2_ flux (%)
Continuously active	1, 2, 4, 9, 10, 11	57.50 ± 2.81	233.42 ± 0.93	1400.51 ± 114.06		~ 74
Regularly active	3, 7, 12, 14	26.61 ± 15.83	108.02 ± 64.27	432.08 ± 35.19		~ 23
Sporadically active	5, 6, 8, 13	3.79 ± 2.17	15.37 ± 8.82	61.48 ± 5.01		~ 3
**Summary**	**1–14**	**33.33 ± 24.78**	**135.29 ± 100.59**	**1894.08 ± 154.26**		**100**
**Extrapolation of CO**_**2**_ **fluxes**						
Reference area			Spatial extent		Estimated CO_2_ flux (t CO_2_ yr^− 1^)	
Area exhibiting pH anomaly ≥ −0.2 units			~ 3,100 m^2^		587.1 ± 47.7 t CO_2_ yr^− 1^	
Area exhibiting pH anomaly ≥ −0.1 units			~ 17,200 m^2^		3257.7 ± 264.9 t CO_2_ yr^− 1^	
Habitat 1180 registered in the Natura 2000 Standard Data Form, SCI ESZZ16010; [10])			0.0684 km^2^		12,993.4 ± 1058,2 t CO_2_ yr^− 1^	
Habitat 1180 updated by LIFE IP INTEMARES project [11]			1.64 km^2^		310,629.1 ± 25,298.6 t CO_2_ yr^− 1^	

### Dissolved inorganic nutrients

**Fig. 5:**
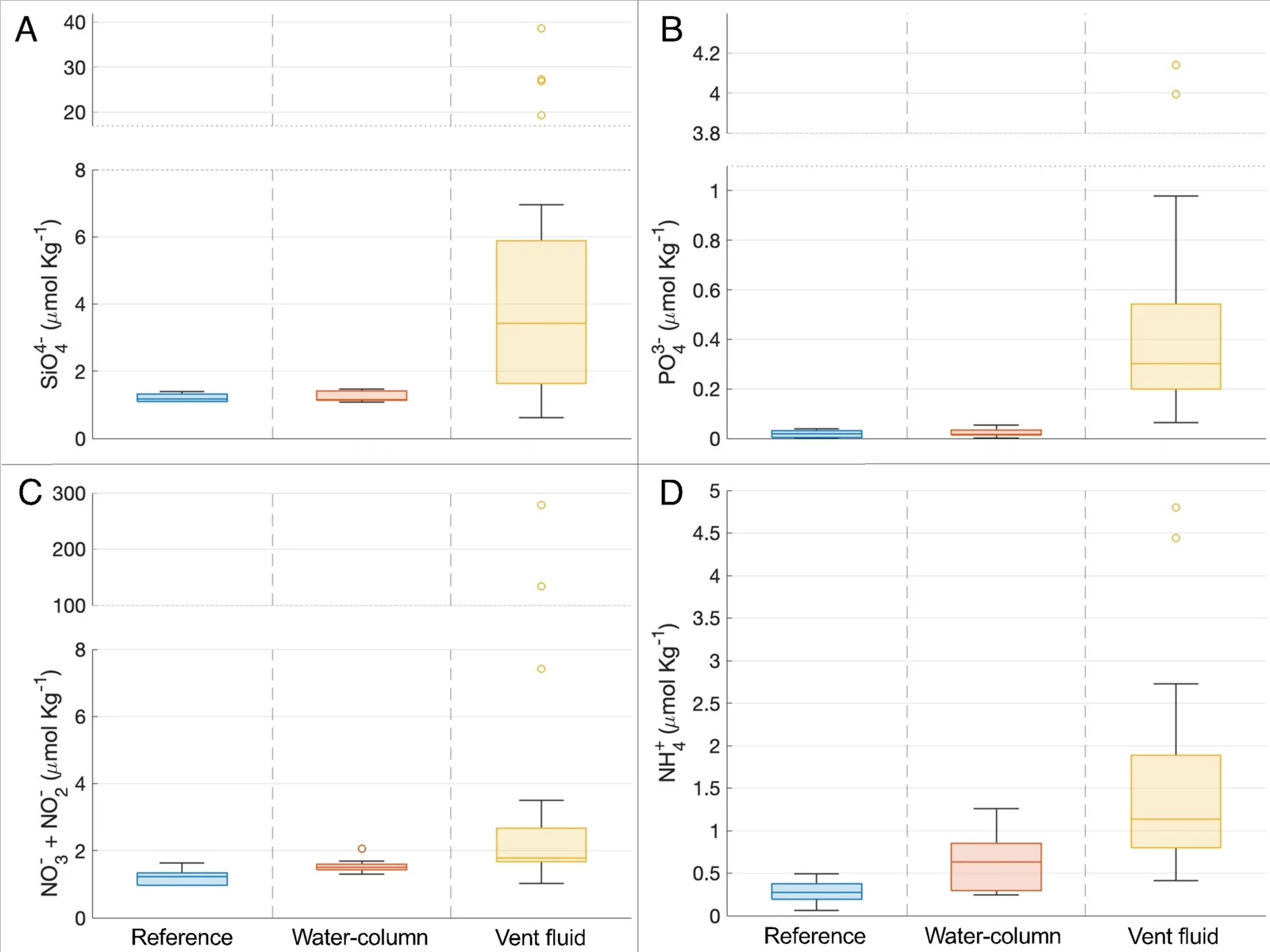
Boxplot of dissolved inorganic nutrient concentrations sorted by the sampling region: Reference as stations away from hydrothermal influence, Water-column as stations located in the water column few meters above the active area, and Vent fluid as stations directly sampled from vents by divers. (**A**) Silicate concentrations; (**B**) Phosphate concentrations; (**C**) Nitrate+nitrite concentrations; (**D**) Ammonium concentrations.

Dissolved inorganic nutrient concentrations differed among reference waters, hydrothermally influenced water-column samples and direct vent fluid samples (Fig. 5 and Supplementary Table S2). Vent fluid samples displayed substantially higher concentrations relative to reference waters, with average enrichments of 5.90-fold for silicate, 50.02-fold for phosphate, 13.86-fold for nitrate+nitrite and 5.61-fold for ammonium. Maximum enrichment factors reached 9.56 for silicate, 89.88 for phosphate, 50.59 for nitrate + nitrite, and 7.07 for ammonium. All vent fluid samples showed significant enrichments in comparison with reference and water-column samples (*p* < 0.05), with the exception of silicate, which only showed significant differences with water-column samples. Reference concentrations averaged 1.21 ± 0.13 µmol kg^− 1^ for silicate, 0.02 ± 0.02 µmol kg^− 1^ for phosphate, 1.23 ± 0.26 µmol kg^− 1^ for nitrate+nitrite and 0.28 ± 0.15 µmol kg^− 1^ for ammonium. In contrast, water-column samples above the vent field showed modest enrichments, with 1.02-fold for silicate, 1.24-fold for phosphate, 1.24-fold for nitrate+nitrite and 2.16-fold for ammonium, showing no significant differences with reference concentrations (*p* > 0.05).

### Microbial community composition

**Fig. 6:**
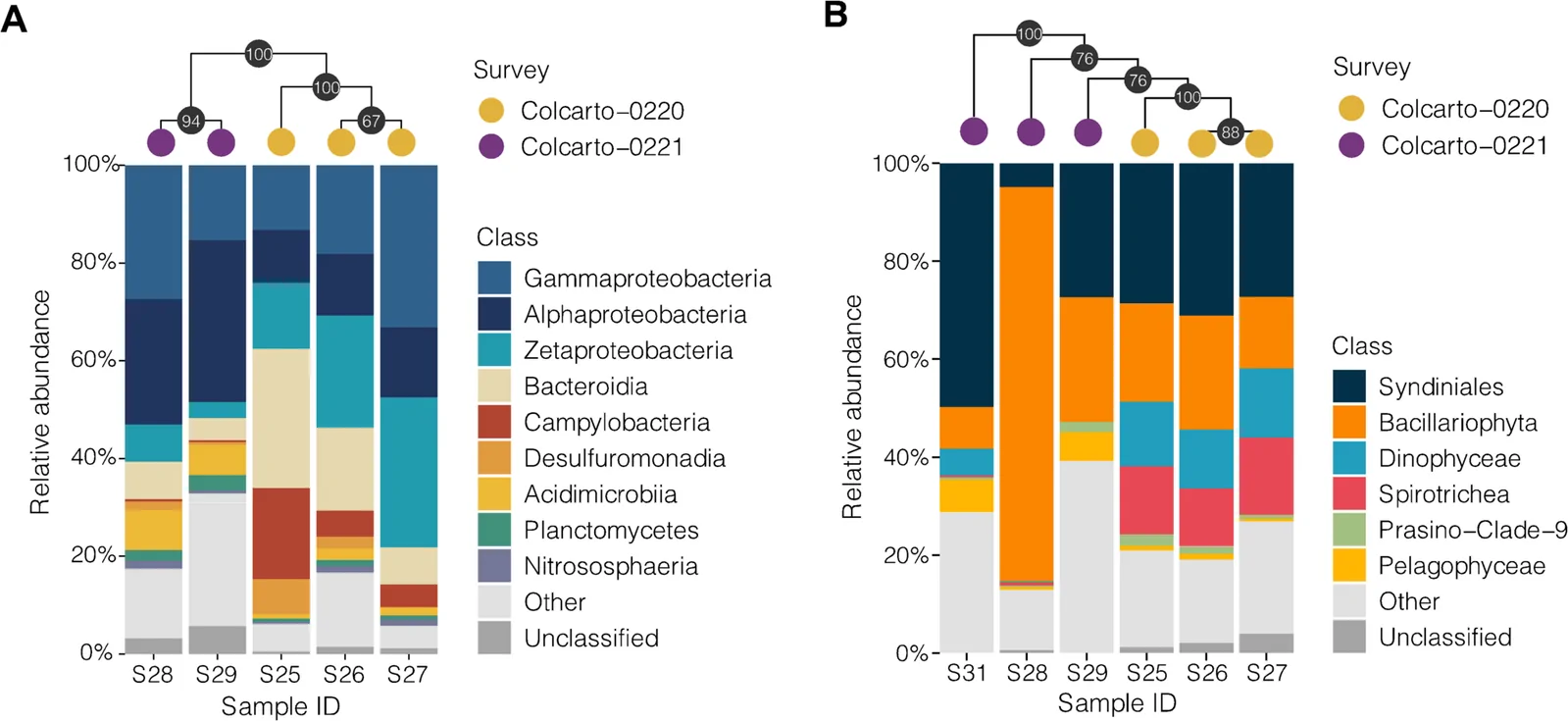
Hierarchical clustering of (**A**) prokaryotic 16 S rRNA and (**B**) eukaryotic 18 S rRNA sequences (excluding Opisthokonta) collected during the 2020 (yellow) and 2021 (purple) surveys, classified. The dendrograms include approximately unbiased (au) values (grey) to indicate clustering uncertainty. Taxa are presented in decreasing abundance, with classes below 1% median relative abundance grouped as “Other”. Note that 2020 survey named Colcarto-0220 and 2021 named Colcarto-0221.

Microbial mat samples collected in 2020 and 2021 exhibited distinct community structures, with hierarchical clustering showing clear separation between years (Fig. 6). While the 2020 samples exhibited lower prokaryotic and higher eukaryotic alpha diversity indices (Chao1 richness and Shannon diversity), the 2021 samples displayed the inverse pattern (Supplementary Fig. S3).

Prokaryotic communities were dominated by Proteobacteria (median relative abundance of 54%), primarily Gamma- (18%), Alpha- (14%), and Zetaproteobacteria (14%). Within Gammaproteobacteria, Colwelliaceae family (4.5%) was prominent, including the genera *Colwellia* (3%) and *Thalassotalea* (1%). Alphaproteobacteria were mainly represented by the Rhodobacteraceae family (8.5%), which included low-abundant genera like the *Roseobacter clade NAC11-7 lineage* (1%). Meanwhile, Zetaproteobacteria, were exclusively represented by the iron-oxidizing genus *Mariprofundus* (Mariprofundaceae family). Other predominant prokaryotic phyla included Bacteroidota (10%), Campylobacterota (5%), Desulfobacterota (3%), and Actinobacteriota (2.5%), each largely dominated by a single taxonomic class (Supplementary Fig. S4). Within Bacteroidia (8%), the Flavobacteriaceae family (2%) was the most abundant, displaying a wide variety of genera. Campylobacteria (5%) were mainly composed of the sulfur-oxidizing Sulfurospirillaceae family (4%) and the *Sulfurospirillum* genus, while the Geopsychrobacteracea family (1%) stood out among the sulfate-reducing Desulfuromonadia (2%). Acidimicrobiia (2.5%) primarily comprised the Microtrichaceae family (1.6%), with most sequences belonging to the *Sva0996 marine group*. Additional low-abundance classes (≤ 1%) included the ammonia-oxidizing archaea Nitrososphaeria (1%) and its family Nitrosopumilaceae (1%).

As noted in the Methods section, the microbial mat samples may include some contribution from allochthonous material derived from the overlying water column. Accordingly, the relatively high proportion of planktonic eukaryotes (dinoflagellates, diatoms) and metazoans (copepods) detected in the sequencing data is consistent with benthic-pelagic coupling in this shallow system and does not necessarily reflect exclusively in situ mat-associated communities. Within prokaryotic communities, taxa with recognized chemolithotrophic roles in hydrothermal environments, including Zetaproteobacteria, Campylobacterota, and Nitrososphaeria, were detected and are consistent with a hydrothermal-associated component of the recovered community.

Eukaryotic communities were initially dominated by Opisthokonta (median relative abundance of 27%), largely represented by metazoans (24%), with Arthropoda (7%) as the most abundant class and mostly consisted of the Maxillopoda family (7%) (Supplementary Fig. S5). After excluding Opisthokonta, the remaining eukaryotic assemblage was dominated by Dinoflagellata (41%) and Ochrophyta (24%) (Supplementary Fig. S6). Dinoflagellate communities were largely composed of Syndiniales, while diatoms such as *Minidiscus*, *Thalassiosira*, and *Navicula* dominated the Ochrophyta. Despite pronounced interannual variability, several prokaryotic and eukaryotic taxa were consistently detected across both surveys.

## Discussion

Our findings reveal a biogeochemically active environment, where subtle spatial and temporal variations in physical-chemical conditions could potentially alter the structure of microbial communities. By comparing these observations with other Mediterranean sites, we provide insights into the processes controlling carbon fluxes and ecosystem responses in a shallow CO_2_-rich environment.

### Local physical-chemical conditions

The physical-chemical variability observed in the water column reflects the combined influence of regional oceanographic dynamics and localized hydrothermal inputs. The short-term and localized changes in temperature and salinity in the upper water column during the 2021 survey appear to be driven by the interaction between seasonal circulation patterns, wind regimes, weather events, and freshwater inputs^12–14^, meanwhile at vent depths, temperature and salinity anomalies remain small, which is consistent with previous observations at Calent^7^, and also with other Mediterranean systems^15,16^. Sediment temperature measurements indicate localized heat release at Calent mound, which rapidly dissipates in the upper meters above the seabed, in agreement with other low-temperature hydrothermal systems in the Mediterranean^17,18^. The decrease in dissolved oxygen concentrations is also observed in other hydrothermal fields, where it is frequently attributed to the oxidation of reduced compounds, such as metals and gases released from venting fluids^19,20^. In addition, negative Brunt-Väisälä frequency values observed at vent depths (Fig. 2) suggest the presence of localized water column instability. While the precise origin of this instability cannot be determined from our data alone, hydrothermal buoyancy forcing and current-topography interactions over Calent mound are both plausible contributing mechanisms. Notably, the co-occurrence of these negative *N*^*2*^ values with independently measured pH reductions and ORP anomalies (geochemical fingerprints of hydrothermal inputs) suggest that venting activity may play a role in driving this instability. Disentangling these processes definitively would require current velocity measurements and turbulent microstructure profiling, which we propose as a priority for future surveys.

To contextualize Calent mound within Mediterranean hydrothermal systems, we observe that vent fluid temperatures in the region range widely. High-temperature systems can reach up to 119 °C^21,22^, while low-temperature systems emit fluids between 13 and 25 °C^4,6,9,22^. Calent mound hydrothermal fluids clearly fall within this lower temperature range, and the modest increase in sediment temperature in comparison with ambient seawater is consistent with sediment temperatures reported from other low-temperature vents^23,24^. However, long-term thermal monitoring would be essential to determine whether Calent mound remains stable or exhibit periodic venting fluctuations, as observed in Milos, with sediment temperatures fluctuating between 24 and 86 °C over an 86-day period^17^ or the seasonal fluctuations attributed to weather conditions during a 1.5-year survey observed in Panarea^24^.

### Spatial extent and intensity of acidification

Among the physical-chemical tracers surveyed, pH proved to be the most sensitive indicator of the hydrothermal activity, enabling detailed mapping of acidic gradients both horizontally and vertically. Regions experiencing pH reductions of at least 0.05 units covered more than 70,000 m², while more intense negative anomalies, reaching up to 1.12 pH units, were confined to smaller near-bottom areas. This pH reduction expands the earlier study at Calent mound^7^, which reported negative pH anomalies averaging 0.2 pH units, with a maximum decrease of 0.6 pH units.

Disentangling the biological effects of CO_2_-driven acidification from those of co-emitted reduced chemical species (e.g., sulfide, ammonium, ferrous iron) remains challenging. At Calent mound, the co-occurrence of pH anomalies with ORP anomalies confirms the simultaneous delivery of both acidifying and reducing compounds to the water column. While the dominant gas phase is CO_2_ and the system falls within the low-temperature hydrothermal category, the presence of chemolithotrophic taxa (Campylobacterota, Zetaproteobacteria, Nitrososphaeria) demonstrates that reduced compounds play a role in shaping microbial community structure. Disentangling CO_2_-specific effects from those of reduced species on biological communities at Calent mound would require comparative data from sites with similar pH anomaly magnitudes but different geochemical compositions (e.g., purely CO_2_-dominated systems with negligible reduced species), which are currently unavailable for this system. We acknowledge this limitation and encourage future geochemical surveys targeting dissolved H_2_S and Fe(II) concentrations, which would allow a more detailed mechanistic interpretation of the relative contributions of acidification and redox chemistry to community structure. Further long-term pH monitoring could determine whether biological impacts are primarily driven by short-term exposure peaks, or by sustained low-pH conditions^14^.

The steep pH gradients delimit discrete areas exposed to near-future and end-of-century ocean acidification scenarios, such as anomalies of −0.1 pH units projected for the seawater surface by 2050 or up to −0.50 pH units predicted by 2100 under high-emission scenarios^2^. Thus, Calent offers a valuable natural setting for investigating biogeochemical and ecological responses to acidification under realistic, fluctuating field conditions. Similar localized acidification patterns have been documented at other Mediterranean CO_2_ vent systems, including Sicily^5^, Vulcano^19^, and Panarea^25^, where sharp pH gradients are maintained by continuous gas release and rapidly returning to normal outside the immediate venting area. At Calent mound, the spatial co-occurrence of pH anomalies with active CO_2_ venting and the dominance of CO_2_ in the gas phase^7^ support CO_2_ dissolution as the primary driver of the observed acidification. However, we acknowledge that discriminating between CO_2_-driven and mineral acid-driven acidification would require discrete water column measurements of total CO_2_ and total alkalinity to apply the ΔCT/ΔpH correlation^26^, which represents a priority for future surveys.

### CO_2_ emissions and venting dynamics

Cyclic emissions have been observed at other submarine hydrothermal systems^27,28^ and have significant ecological implications^17^, as venting fluctuations influence local physical-chemical dynamics. Gas emissions at Calent mound are characterized by strong heterogeneity in both temporal behavior and flux intensity. While some vents exhibit continuous degassing, others display periodic bubbling with variable emission frequencies. Despite this variability, continuously active vents dominate the overall CO_2_ output, accounting for nearly three-quarters of the total emissions within the surveyed area.

Estimated CO_2_ flux rate at Calent mound (189.4 ± 15.4 kg CO_2_ m^− 2^ yr^− 1^) exceeds values reported for several other shallow hydrothermal systems worldwide^29^. Spatially scaled extrapolations are summarized in Table 1 and discussed here in terms of their underlying assumptions and uncertainties. The pH anomaly-based estimates (587–3258 t CO_2_ yr^− 1^) are directly estimated from geochemical observations at Calent mound, but may be affected by hydrodynamic dispersion and lateral plume drift. The estimate based on the active vent field cartography in the Natura 2000 Standard Data Form for SCI ESZZ16010 (12993 ± 1056 t CO_2_ yr^− 1^; Data Quality: Good) provides an independent, institutionally validated spatial reference and directly comparable in scale to other shallow water hydrothermal systems^29^. The upper-bound estimate based on the updated extent of Habitat 1180 mapped by the LIFE IP INTEMARES project (310,616 ± 25,256 t CO_2_ yr^− 1^) encompasses gas-emitting areas beyond Calent mound^11^, including zones south of the mound, and should therefore be interpreted as a system-wide theoretical maximum for the entire Columbretes archipelago rather than a Calent mound-specific value.

All extrapolation estimates assume spatial homogeneity of flux density across the reference areas. This assumption is unlikely to hold given the documented heterogeneity in vent activity at Calent mound, where continuously active vents account for ~ 74% of total emissions despite representing only ~ 42% of observed vents. Additionally, estimates are based on a single 5-hour video recording, and seasonal or episodic fluctuations in degassing intensity, could significantly affect annual totals. Together, these considerations indicate that the reported values should be interpreted as order-of-magnitude estimates, and that future high-resolution spatial surveys combining hydroacoustic bubble detection and long-term pH monitoring are needed to achieve more robust and spatially explicit flux quantification^18,30^ across the full extent of the Columbretes Islands.

It is worth noting that the Habitat 1180 extent currently registered in the Natura 2000 Standard Data Form for SCI ESZZ16010^10^ predates the comprehensive cartographic surveys conducted under the LIFE IP INTEMARES project, and is therefore expected to be updated in a future SDF release to reflect the substantially larger extent of 164 ha^11^. This discrepancy underscores the importance of integrating outputs from dedicated scientific surveys into institutional biodiversity databases in a timely manner, particularly for habitats such as Habitat 1180 whose spatial extent has direct implications for conservation management and emissions assessments under the EU Habitats Directive.

### Nutrient enrichment associated with hydrothermal fluids

Hydrothermal fluids at Calent mound are significantly enriched in dissolved inorganic nutrients, despite previous measurements reporting no significant differences between control and vent stations^7^. A direct comparison with this previous study is not possible since the precise sampling locations were not reported. The presence of ammonia-oxidizing organisms suggest that ammonium is relevant in vent fluids, which gradually is oxidized to nitrite and then nitrate, increasing their concentrations, as also reported in other hydrothermal systems^31,32^.

Nutrient enrichment associated with hydrothermal activity has been reported for other CO_2_-vented systems^5,25,31^, where a rapid decrease of concentrations has also been also observed, limiting their spatial scale of influence. Nevertheless, the strong enrichment at the vent source but rapid dilution in the water column at Calent mound, indicates biogeochemical impacts are spatially restricted yet potentially intense within the immediate venting zone.

### Microbial community composition

Prokaryotic and eukaryotic community structures display interannual shifts, emphasizing the complexity of the communities associated with benthic microbial mats inhabiting the shallow hydrothermal system of Calent mound, enriched in nutrients and reduced compounds within the photic zone. In this setting, CO_2_ is fixed using energy derived either from sunlight or from the oxidation of the reduced compounds^33,34^. Accordingly, microbial community structure is likely shaped by the interplay of physical-chemical gradients, biogeochemical fluxes, and biological interactions^17,25^.

It is important to note that the microbial mat samples likely integrate a mixture of hydrothermal and background marine signals. While taxa such as Zetaproteobacteria (iron-oxidizing *Mariprofundus*), Campylobacterota (sulfur-oxidizing Sulfurospirillaceae), and ammonia-oxidizing Nitrososphaeria are consistent with hydrothermal chemolithotrophic processes linked to the geochemical fluxes of reduced iron, sulfur compounds, and ammonium^36–38^, other abundant groups, such as Rhodobacteraceae within Alphaproteobacteria and Actinobacteriota, are widespread in non-hydrothermal marine environments and likely represent background marine communities co-occurring in the mat. Similarly, the presence of planktonic and metazoan eukaryotes (dinoflagellates, diatoms, copepods) suggests a significant contribution from the overlying water column, consistent with benthic-pelagic coupling in a shallow, photic-zone system. These mixed signals limit the extent to which community composition can be attributed exclusively to hydrothermal activity, particularly for generalist marine taxa, and highlight the need for non-hydrothermal reference samples from nearby sites in future studies to strengthen ecological interpretations. In this study we focus on describing the composition and interannual variability of the communities present, acknowledging that the relative contribution of hydrothermal versus background signals cannot be fully disentangled with the currently available dataset.

Within eukaryotic assemblages, the high relative abundance of copepod sequences in the 18 S dataset may reflect benthic-pelagic coupling and input from the overlying water column, consistent with the shallow photic-zone setting, rather than necessarily indicating elevated in situ copepod densities driven by hydrothermal productivity. It should be noted that the sequencing data are relative (compositional) rather than quantitative; they cannot be used to make absolute abundance claims without paired biomass or density measurements. Nonetheless, the dominance of copepod-associated sequences is consistent with observations from other shallow hydrothermal systems where enhanced primary production driven by hydrothermal nutrient inputs supports elevated metazoan abundances in the water column above active vents, such as the Tagoro submarine volcano in the Canary Islands^39^. Similarly, the occurrence of Syndiniales (parasitic dinoflagellates) highlights their ecological relevance in hydrothermal systems, consistent with findings from both deep and shallow hydrothermal systems, where the high productivity of the region associated with hydrothermal activity increases host and prey biomass and thereby, parasitic encounters and infections^39,40^.

The presence of centric diatoms reinforces the idea of elevated surface productivity associated with shallow hydrothermal systems, where the light availability and nutrient enrichment from hydrothermal inputs may locally support photosynthetic planktonic organisms. While fluorescence profiles obtained by the CTD-mounted fluorescence sensor showed no marked enhancement directly above the vent field relative to reference stations (Supplementary Fig. S7), this likely reflects the highly localized nature of the nutrient inputs and their rapid dilution into the water column, consistent with the modest enrichments observed for water-column nutrient samples. It should be noted that hydrothermal inputs do not universally stimulate photosynthesis; in some systems they can suppress it through shading by turbid plumes or through toxic metal inputs^33^ At Calent mound, the low-temperature character of the venting and the absence of detectable reduced metal enrichments in the water column suggest that stimulatory effects are more plausible here, though this needs further investigation.

Together, these data underscore the ecological complexity of benthic microbial mat communities in shallow hydrothermal systems. The fluctuating pH levels at Calent mound offer a dynamic natural environment to assess individual, population and community-level responses in ways that laboratory experiments with constant pH conditions cannot^14^. The benthic microbial mat communities at Calent mound show both persistent core taxa and dynamic shifts in relative abundance between survey years, driven by hydrothermal activity and associated geochemical gradients; water-column microbial communities, however, are expected to be more variable, but they are not the focus of our compositional analysis. Long-term monitoring is needed to fully characterize these communities and understand the temporal dynamics of community structure and species interactions^18,35^.

### Calent mound within the spectrum of shallow-water hydrothermal systems

Within the spectrum of shallow-water hydrothermal environments described in the Mediterranean^34^, between purely CO_2_-dominated vent systems^4^ and more geochemically complex hydrothermal fluid systems^9^, Calent mound is most appropriately positioned toward the CO_2_-dominated systems: its gas phase is dominated by CO_2_^7^, vent fluid temperatures fall in a low-temperature range, and pH anomalies return rapidly to background values outside the venting area, which is consistent with patterns documented at analogous systems in Sicily, where CO_2_ also dominates at 98.1% with only trace H_2_S^4^, and in Milos, where CO_2_-rich degassing drives localised pH reductions in the water column^9^. This CO_2_-dominated character is further supported by the prior characterization of this same system^7^, who focused exclusively on CO_2_ and carbonate chemistry with no detection of sulfur in the gas. However, the significant enrichment of vent fluids in dissolved inorganic nutrients, co-occurring ORP anomalies, and the presence of a chemolithotrophic microbial community sustained by sulfur, iron, and ammonia-oxidising taxa indicate that reduced chemical species also contribute to the biogeochemistry of Calent mound, placing it beyond a purely gas-dominated system. The consistent detection of these chemolithotrophic taxa across both survey years thus provides biological evidence for the availability of reduced substrates at the vent-sediment interface, and dedicated in situ measurements, including H_2_S, H_2_, and dissolved iron, represent a priority for future work to directly couple fluid composition with microbial metabolic pathways.

### Methods

#### Study area

The hydrothermal field lies at Calent mound, in the Columbretes archipelago, located ~ 55 km off the coast of Castellón (western Mediterranean), at depths ranging from 35 to 51 m (Fig. 1). Hydrographic surveys, seawater and gas samples were collected in two oceanographic surveys (Colcarto-0220 in 2020 and Colcarto-0221 in 2021) to characterize the physical, chemical and biological properties of this shallow CO_2_ vent system.

#### Hydrographic survey

Hydrographic profiles were acquired using a SeaBird 911-plus CTD, recording conductivity, temperature and pressure data at a sampling frequency of 24 Hz, with accuracies of 0.001 °C for temperature and 0.0003 S m^− 1^ for conductivity. A SBE27 pH-ORP sensor (accuracy of ± 0.02 pH units) was integrated into the CTD package and factory-calibrated by Sea-Bird Scientific prior to each cruise, and verified onboard using buffer solutions (pH 4, 7 and 10). Fluorescence was recorded using a WET Labs ECO-AFL/FL sensor (precision ± 0.01 mg m^− 3^). The dissolved oxygen concentrations recorded in ml L^− 1^ by the SBE43 (precision ± 2% of saturation), were converted to µmol kg^− 1^ using in situ potential density derived from the CTD data. The Brunt-Väisälä frequency (*N*^*2*^) was calculated using the density profile of the water column to asses water column stability. Positive *N*^*2*^ values indicate stable stratification, values near zero indicate weak stratification or mixing, and negative values indicate instability. Anomaly of pH (ΔpH) was calculated as the difference between hydrothermally influence stations and reference stations. The rate of change between consecutive measurements of ORP (δORP) is used as a proxy to identify sharp redox transitions associated with hydrothermal venting, as absolute ORP values are subject to sensor drift and baseline offsets.

Multiple survey strategies were employed to obtain a comprehensive characterization of the seawater column properties (Fig. 1B). Discrete vertical profiles (VP) were conducted to assess the vertical structure of the seawater column. In addition, high-resolution spatial sampling was achieved through continuous transects using two distinct methodologies: (i) near-seabed tows (NS), where the CTD was maintained 2–3 m above the seafloor while the vessel advanced (~ 0.4 kt; ~0.21 m s^− 1^), focusing mainly on hydrothermally influenced bottom waters; and (ii) tow-yo transects (TW), consisting of repeated vertical movements (from surface to 2–3 m above the seabed) while the vessel moved slowly (~ 0.4 kt), creating a sawtooth pattern dataset. Reference stations (far-field sites) located outside the active hydrothermal area were also sampled for comparison with hydrothermally influenced stations. In total, 8 hydrographic stations were surveyed within the hydrothermal field (6 in 2020 and 2 in 2021), with 5 NS, 2 TW and 1 VP, along with 8 reference stations located away from the venting area (5 in 2020 and 3 in 2021), comprising 6 NS and 2 TW (Supplementary Table S8). Horizontal distribution of pH shown in Fig. 3 was mapped from NS20 01, NS20 02, NS20 06, NS20 07, while vertical distribution was mapped from TW21 38.

In 2021, in situ temperature measurements were also obtained directly from hydrothermal vents using HOBO Water Temperature Pro v2 Data Loggers (U22-001). Sensors were deployed by scuba divers in bubbling areas at three different depths: 20 cm into the sediment, at the sediment surface and 20 cm above the seafloor. Temperature data were recorded at 10 s intervals over a 4-day period.

#### Gas sampling and CO_2_ flux estimation

Gas samples from vents were collected during the 2021 cruise to determine CO_2_ concentration in the emitted gas phase (Fig. 1B-C). Eight samples were collected by divers using an inverted funnel connected to 12 mL glass tubes capped with butyl rubber septa and stored at room temperature until analysis. The free gas was analyzed using cavity ring-down spectroscopy on a Picarro G2201i (Picarro Inc., Santa Clara, USA) coupled to an Automate™ gas sampler through a Picarro Liason system, using zero air as carrier gas. The instrument response to CO_2_ concentrations was calibrated against standards produced by acidifying weighed amounts of solid CaCO_3_ with 3 mL of 10% phosphoric acid in gas-tight tubes (identical to those used for taking the samples). Air in the standard tubes was purged for 1 min with zero air through the septum prior to acidification to remove ambient CO_2_; because each mole of CaCO_3_ stoichiometrically yields one mole of CO_2_, the calibration relates the instrument signal directly to the absolute amount of CO_2_ contained in a tube of fixed volume (12 mL, linear calibration, r² = 0.9986). The reported CO_2_ concentrations (mol L^− 1^) therefore correspond to the free gas emitted at the vent orifice, expressed as moles of CO_2_ per litre of collected gas at the in situ sampling conditions (5.3 atm, 18.08 °C). These values expressed as moles per litre allow direct calculation of molar CO_2_ fluxes from the gas outflow rate (mL s^− 1^) measured in situ. The CO_2_ content of the gas bubbles could not be directly compared to, or used to infer, dissolved inorganic carbon concentration in the surrounding seawater, as equilibration between the gas and liquid phases could not be assumed. The emitted bubbles likely retained a composition reflecting source conditions rather than having exchanged CO_2_ with the ambient water column. Therefore, the pH anomalies reported in this study were measured independently in situ using calibrated CTD sensors, and were not derived from the gas-phase concentration data. Dissolved inorganic carbon parameters (DIC, total alkalinity, seawater pCO_2_) were not measured, which we acknowledge as a limitation for fully constraining the carbonate system.

CO_2_ flux per vent was calculated based on gas outflow velocity (mL s^− 1^), CO_2_ concentration (mol L^− 1^) and emitting period (min h^− 1^). Gas outflow rate (mL s^− 1^) was estimated by measuring the time required to fill a 12 mL tube at five different vents (mean filling time of 6.4 ± 0.5 s). Emitting periods in cyclic vents was estimated by calculating the duration of no-emitting periods between bubbling pulses. To address this issue, we used the cumulative duration of bubble emissions per hour of video footage to estimate active degassing time. In addition, extrapolation of CO_2_ fluxes to a broader area was calculated by using the average annual CO_2_ flux per unit of area estimated from the ~ 10m^2^ study area (Table 1).

Temporal variability and potential cyclicity in gas bubbling activity were assessed using high-resolution video footage acquired during 2021 cruise. Continuous recordings (5 h duration) were obtained using a SONY HDR-AS50 camera, capturing an area of ~ 10 m^2^ encompassing 14 bubbling vents (Fig. 4A). The duration of individual gas emissions was visually measured and analyzed using two complementary approaches. One of them is the Fast Fourier Transform (FFT) to evaluate the signal power content at different frequencies^41–43^. This widely used technique is designed for stationary signals, however, many geophysical signals contain non-stationary or transitional characteristics: trends, abrupt changes, drift, etc., and FFT is not suited for their detection. For this reason, we also applied Wavelet Transform (WT) analysis, which provides the temporal location of the frequencies within the time series^43–46^.

Supplementary Table S1 contains in details the significant periodicities obtained from FFT and estimated fluxes for each vent.

#### Dissolved inorganic nutrient analysis

Samples were collected from reference and water column above the active area using an oceanographic rosette equipped with 12 5-L Niskin bottles during both cruises. In addition, hydrothermal fluids were sampled directly from vent emissions by scuba divers using 12 mL syringes. A total of 55 seawater samples were collected, with 6 samples from reference stations (Reference in Fig. 5), 25 from hydrothermally influenced waters (CTD in Fig. 5), and 24 from direct vent emissions (Dives in Fig. 5). Concentrations and enrichment ratios for each station can be found as Supplementary Table S2. Nutrient samples were immediately frozen at −20 °C until analysis. Concentrations of nitrite + nitrate (NO_2_^–^ + NO_3_^–^), phosphate (PO_4_^3–^), silicate (Si(OH)_4_) and ammonium (NH_4_^+^) were determined using a five-channel automatized Technicon-Bran Luebbe AA III AutoAnalyzer following colorimetric methods. Analytical quality control was performed for each nutrient using standards prepared by pipetting appropriate volumes of OSIL certified reference solutions (concentrations of: silicate 1000 µmol L^− 1^, phosphate 100 µmol L^− 1^, nitrate 1000 µmol L^− 1^, nitrite 100 µmol L^− 1^, and ammonium 10 mmol L^− 1^; all ± 1% of associated uncertainties) into 50 mL volumetric flasks and making up to volume with ultrapure water, yielding target concentrations of 20 µmol L^− 1^ for silicate, 1 µmol L^− 1^ for phosphate, 20 µmol L^− 1^ for nitrate, 1 µmol L^− 1^ for nitrite, and 6 µmol L^− 1^ for ammonium. The theoretical concentration of each working standard, including the combined uncertainty from pipette volume, volumetric flask, and reference material concentration, defined a theoretical concentration interval. Each working standard was measured in triplicate on the autoanalyzer; the resulting mean and standard deviation were used to define a 95% confidence interval for the measured concentration. Quality control was considered satisfactory when the measured concentration interval fell within the theoretical interval. When this criterion was not met, a correction factor was calculated as the ratio of the theoretical to the measured mean concentration and applied to all sample measurements from that analytical run. Analytical precision, estimated as the standard deviation of three consecutive measurements of the working standards, was: 0.0340 µmol L^− 1^ for silicate, 0.0016 µmol L^− 1^ for phosphate, 0.0992 µmol L^− 1^ for nitrate, 0.0007 µmol L^− 1^ for nitrite, and 0.0064 µmol L^− 1^ for ammonium. Nutrient concentrations, originally determined in µmol L^− 1^, were subsequently converted to µmol kg^− 1^ using the in situ seawater density measured individually for each sample.

Nutrient enrichment was calculated as the ratio of nutrient concentration in hydrothermally influenced samples (water column and vents fluids) to those measured at reference stations. Statistical analyses were performed to evaluate significant differences between reference, hydrothermally influenced samples and vent fluids, using Kruskal-Wallis test. Pairwise comparisons were performed with Bonferroni correction for multiple testing. Analyses were performed in Matlab R2021a.

#### Microbial mat sampling and sequencing

Six microbial mat samples were collected from the active hydrothermal field (Fig. 1B-C, Supplementary Table S9) to characterize local microbial communities, including archaea, bacteria, and eukaryotes, using sequencing techniques. The term “microbial mat” is used here to describe the macroscopically visible, dark-colored, gelatinous biofilm material visually identified by divers directly overlying the hydrothermal vent orifices (Fig. 1C). Scuba divers collected this surface-associated material using sterile 15 mL Falcon tubes, and samples were preserved at −20 °C until analysis. Given the shallow water nature of the system and its direct exposure to the overlying water column, some contribution from settling pelagic organisms or resuspended sediment particles cannot be excluded.

Genomic DNA extraction and sequencing library preparation were performed at the Oceanographic Center of the Canary Islands (IEO-CSIC). DNA was extracted from mat samples using the OmniPrep™ Soil DNA kit (G-Biosciences, Saint Louis, MO, USA) following the manufacturer’s instructions. The V4 region of the prokaryotic 16 S rRNA (~ 390 bp) was amplified using primers 515 F (GTGYCAGCMGCCGCGGTAA)^47^ and 806R (GGACTACNVGGGTWTCTAAT)^48^, while the V4 region of the eukaryotic 18 S rRNA (~ 260 ± 50 bp) was targeted using the TAReuk454FWD1 (CCAGCASCYGCGGTAATTC) and TAReukREV3 (ACTTTCGTTCTTGATYRA) primer set^49^. The sequencing libraries were prepared following the procedure described in^36^. Cleaned and indexed samples were paired-end sequenced (2 × 300 bp) on an Illumina MiSeq platform (Macrogen, Korea). Raw sequences are available in the NCBI public database under the accession number PRJNA1168318.

Primers and adapters were removed from the sequences using cutadapt v.1.18^50^. The DADA2 v1.28 pipeline^51^ was computed with filtering parameters derived from FIGARO analyses^52^. Cleaned sequences were trimmed at *truncLen* 216,143 with *maxEE* 1 for the prokaryotic dataset, and at *truncLen* 223,200 with *maxEE* 2 for the eukaryotic dataset. Sample inference was performed by pooling all samples together, followed by chimera removal using the consensus approach. An amplicon sequence variant (ASV) table was obtained for each sequenced gene. Taxonomic assignment for prokaryotic sequences was conducted according to Silva SSU version 138.1, while eukaryotic taxonomy was assigned using the PR^2^ database^53^. Amplicon datasets were rarefied to avoid differences in sequencing depth (Supplementary Table S9) using phyloseq v.1.44^54^. Sequences from the eukaryotic supergroup Opisthokonta (~ 27% median relative abundance among eukaryotic supergroups across all samples) were analyzed separately in this study. This group includes a great diversity of animals and heterokonts that may benefit from microbial mats rather than representing the dominant organisms inhabiting them.

A total of 350,667 prokaryotic 16 S rRNA reads were analyzed, resulting in 3,316 amplicon sequence variants (ASVs) with a median of 62,142 reads per sample (Supplementary Table S9). To standardize the sampling effort and minimize differences in microbial diversity, the prokaryotic dataset was normalized to a minimum sequencing depth of 36,709 reads, which led to the loss of 331 ASVs. In parallel, the analysis of 215,468 eukaryotic 18 S rRNA reads yielded 2,063 ASVs with a median of 30,285 reads per sample (Supplementary Table S9). The eukaryotic dataset was similarly normalized to 27,358 reads, resulting in 1,941 ASVs, of which 270 belonged to the Opisthokonta supergroup.

Alpha diversity was assessed using the Chao1 and Shannon diversity metrics estimated through phyloseq v.1.44. To evaluate differences in microbial community structure among samples, multiscale bootstrap resampling was conducted using pvclust v2.2^55^ in hierarchical cluster analysis based on Bray-Curtis dissimilarities, which computed approximately unbiased probabilities (au) for each cluster. All microbial analyses were performed in R v4.3.1 and analyzed using tidyverse v2.0.

## Supplementary Information

Below is the link to the electronic supplementary material.


Supplementary Material 1


## Data Availability

The sequencing data generated and analyzed in this study have been deposited in the NCBI database and are publicly available under accession number PRJNA1168318. All other data supporting the findings of this study are provided within the article and its Supplementary Material files.
