# Supplementary material for: Physical-chemical gradients, CO2 venting dynamics and microbial community composition in a shallow Mediterranean CO2-rich hydrothermal system
Source: Sci Rep. 2026 Jun 13;16:27061. doi: 10.1038/s41598-026-57265-z (PMC13522445; doi:10.1038/s41598-026-57265-z)
Supplement: Supplementary file 1 [file 41598_2026_57265_MOESM1_ESM.docx]

**Physical-chemical gradients, CO_2_ venting dynamics and microbial community composition in a shallow Mediterranean CO_2_-rich hydrothermal system**

Juan Pablo Martín-Díaz^1-2^, Alba González-Vega^1^, Clàudia Pérez-Barrancos^1^, Jesús M. Arrieta^1^, Desirée Palomino^3^, Ignacio Baena-Vega^4^, Sandra Mallol^4^, Nuria R. de la Ballina^4^, Irene Díez^5^, Juan T. Vázquez^3^, David Díaz^4^, Eugenio Fraile-Nuez^1^*

^1^ Centro Oceanográfico de Canarias, Instituto Español de Oceanografía, Consejo Superior de Investigaciones Científicas (IEO-CSIC), 38180 Santa Cruz de Tenerife, Spain. [juanpablo.martin@ieo.csic.es](mailto:juanpablo.martin@ieo.csic.es), [alba.gonzalez@ieo.csic.es](mailto:alba.gonzalez@ieo.csic.es), [claudia.perez@ieo.csic.es](mailto:claudia.perez@ieo.csic.es), [jesus.arrieta@ieo.csic.es](mailto:jesus.arrieta@ieo.csic.es), [Eugenio.fraile@ieo.csic.es](mailto:Eugenio.fraile@ieo.csic.es).

^2^ Universidad de La Laguna (ULL), 38200 San Cristóbal de La Laguna, Spain

^3^ Centro Oceanográfico de Málaga, Instituto Español de Oceanografía, Consejo Superior de Investigaciones Científicas (IEO-CSIC), 29002 Málaga, Spain. [desiree.palomino@ieo.csic.es](mailto:desiree.palomino@ieo.csic.es), [juantomas.vazquez@ieo.csic.es](mailto:juantomas.vazquez@ieo.csic.es).

^4^ Centro Oceanográfico de Baleares, Instituto Español de Oceanografía, Consejo Superior de Investigaciones Científicas (IEO-CSIC), 07015 Mallorca, Spain. [Ignacio.baena@ieo.csic.es](mailto:Ignacio.baena@ieo.csic.es), [Sandra.mallol@ieo.csic.es](mailto:Sandra.mallol@ieo.csic.es), [nuria.rodriguez@ieo.csic.es](mailto:nuria.rodriguez@ieo.csic.es), [david.diaz@ieo.csic.es](mailto:david.diaz@ieo.csic.es).

^5^ Servicios Centrales, Instituto Español de Oceanografía, Consejo Superior de Investigaciones Científicas (IEO-CSIC), 28002 Madrid, Spain. [Irene.diez@ieo.csic.es](mailto:Irene.diez@ieo.csic.es)

*corresponding autor: Eugenio Fraile-Nuez (eugenio.fraile@ieo.csic.es)

**Supplementary Table S1.** Gas emission frequency results from 11 vents studied along 5-hour survey and gas fluxes estimates during Colcarto-0221 in 2021. Vents with continuous emissions (vent 4, 10 and 11) are not included. CO_2_ fluxes are calculated from CO_2_ concentration (mol CO_2_ L^-1^) and average velocity outflow (mL h^-1^) and then, units were converted to kg CO_2_ yr^-1^ using the molecular mass of CO_2_ (44 g mol^-1^).

| **Id Vent** | **Range of significant frequency [Hz]** | | | **Range of significant periods [s]** | | | **Time span of emissions** | | | **CO_2_ fluxes** |
| --- | --- | --- | --- | --- | --- | --- | --- | --- | --- | --- |
|  | **Min Freq [Hz]** | **Max Freq [Hz]** | **Most significative [Hz]** | **Min Period [s]** | **Max period [s]** | **Most significative [s]** | **Total video (h)** | **Time of emissions (h)** | **Emissions (min) / h of video** | **(kg CO_2_ yr^-1^ ± SD)** |
| **Vent 1** | 0.00588 | 0.00647 | 0.01241 | 154.65 | 170.11 | 80.56 | 5.08 | 4.72 | 55.75 | 226.31 ± 18.43 |
|  | 0.01888 | 0.02421 |  | 41.30 | 52.97 |  |  |  |  |  |
| **Vent 2** | 0.00770 | 0.01661 | 0.04652 | 60.21 | 129.95 | 21.49 | 5.14 | 4.74 | 55.36 | 224.72 ± 18.30 |
|  | 0.03980 | 0.05199 |  | 19.24 | 25.12 |  |  |  |  |  |
| **Vent 3** | 0.03564 | 0.09773 | 0.04051 | 10.23 | 28.06 | 24.68 | 3.61 | 1.08 | 18.03 | 73.18 ± 5.96 |
| **Vent 5** | 0.01401 | 0.06032 | 0.02440 | 16.58 | 71.36 | 40.98 | 5.15 | 0.23 | 2.73 | 11.07 ± 0.90 |
|  | 0.07073 | 0.08103 |  | 12.34 | 14.14 |  |  |  |  |  |
| **Vent 6** | 0.01470 | 0.04589 | 0.03262 | 21.79 | 68.02 | 30.66 | 3.00 | 0.34 | 6.78 | 27.52 ± 2.24 |
| **Vent 7** | 0.00104 | 0.00514 | 0.00117 | 194.64 | 957.85 | 856.42 | 5.06 | 1.03 | 12.20 | 49.52 ± 4.03 |
| **Vent 8** | 0.00151 | 0.01963 | 0.00436 | 50.94 | 661.26 | 229.38 | 5.10 | 0.33 | 3.87 | 15.72 ± 1.28 |
| **Vent 9** | 0.00323 | 0.00803 | 0.00362 | 124.55 | 309.78 | 275.88 | 5.08 | 4.56 | 53.89 | 218.77 ± 17.82 |
| **Vent 12** | 0.03274 | 0.04530 | 0.03274 | 22.08 | 30.55 | 30.55 | 2.73 | 2.19 | 48.27 | 195.93 ± 15.96 |
|  | 0.07449 | 0.07556 |  | 13.23 | 13.42 |  |  |  |  |  |
| **Vent 13** | 0.01642 | 0.07840 | 0.04121 | 12.76 | 60.89 | 24.26 | 4.77 | 0.14 | 1.77 | 7.18 ± 0.59 |
| **Vent 14** | 0.01834 | 0.04026 | 0.02276 | 24.84 | 54.53 | 43.94 | 2.61 | 1.22 | 27.95 | 113.46 ± 9.24 |
|  | 0.12892 | 0.14404 |  | 6.94 | 7.76 |  |  |  |  |  |

**Supplementary Table S2.** Results of dissolved inorganic nutrient concentrations in waters influenced by the hydrothermal activity and at reference stations. Samples were collected from the water column above hydrothermal vents and directly from vent emissions. Concentrations are given in µmol Kg^-1^. Enrichment is showed as enrich.

| **Cruise**  **(year)** | **Location** | **Station** | **Latitude**  **(Nº)** | **Longitude**  **(Eº)** | **Depth (m)** | **pH** | **SiO_4_^4-^** | **SiO_4_^4-^ enrich.** | **PO_4_^3-^** | **PO_4_^3-^ enrich.** | **NO_3_ + NO_2_** | **NO_3_ + NO_2_ enrich.** | **NH_4_^+^** | **NH_4_^+^ enrich.** |
| --- | --- | --- | --- | --- | --- | --- | --- | --- | --- | --- | --- | --- | --- | --- |
| Colcarto-0220  (2020) | Calent mound | VP20 05 | 39.83528 | 0.68154 | 40-43 | 7.77 ± 0.11 | 1.43 ± 0.04 | 1.05 | 0.04 ± 0.01 | 1.17 | 1.67 ± 0.15 | 1.19 | 0.28 ± 0.03 | 1.08 |
|  |  | D20 01 | 39.83548 | 0.68205 | 40 | - | 0.77 ± 0.20 | 0.56 | 0.34 ± 0.32 | 9.65 | 2.60 ± 2.39 | 1.86 | 1.35 ± 0.45 | 5.20 |
|  |  | D20 02 | 39.83592 | 0.68205 | 40 | - | 13.04 ± 13.80 | 9.56 | 0.37 ± 0.33 | 10.65 | 70.64 ± 114.29 | 50.59 | 0.75 ± 0.26 | 2.90 |
|  | Ref. station | NS20 08 | 39.8584 | 0.71990 | 35-45 | 8.17 ± 0.00 | 1.36 ± 0.05 | - | 0.04 ± 0.01 | - | 1.40 ± 0.35 | - | 0.26 ± 0.10 | - |
| Colcarto-0221  (2021) | Calent mound | NS20 09 | 39.83492 | 0.68292 | 51-52 | 8.52 ± 0.06 | 1.14 ± 0.01 | 1.01 | 0.02 ± 0.01 | 1.48 | 1.47 ± 0.07 | 1.28 | 0.89 ± 0.24 | 3.06 |
|  |  | TW21 38 | 39.83472 | 0.68108 | 37-41 | 7.94 ± 0.22 | 1.13 ± 0.03 | 1.00 | 0.01 ± 0.01 | 1.08 | 1.44 ± 0.08 | 1.25 | 0.72 ± 0.20 | 2.49 |
|  |  | D20 03, 05, 07 | 39.83512 | 0.68208 | 40 | - | 7.63 ± 9.16 | 6.74 | 0.98 ± 1.45 | 89.88 | 1.71 ± 0.12 | 1.49 | 2.05 ± 1.51 | 7.07 |
|  | Ref. station | NS20 14, VP21 48 | 39.83086  39.93608 | 0.67606  0.60964 | 37-51 | 8.68 ± 0.15 | 1.13 ± 0.04 | - | 0.01 ± 0.01 | - | 1.15 ± 0.21 | - | 0.29 ± 0.19 | - |
| **Summary** | | | | | | | | | | | | | | |
| **Reference ^a^** | | | | | | 8.51 ± 0.29 | 1.21 ± 0.13 | - | 0.02 ± 0.01 | - | 1.23 ± 0.26 | - | 0.28 ± 0.15 | - |
| **Water-column ^b^** | | | | | | 8.07 ± 0.36 | 1.24 ± 0.15 | 1.02 | 0.02 ± 0.02 | 1.24 | 1.53 ± 0.15 | 1.24 | 0.61 ± 0.32 | 2.16 |
| **Vent fluid ^c^** | | | | | | - | 7.27 ± 10.07 | 5.90 | 0.67 ± 1.07 | 50.02 | 19.16 ± 61.34 | 13.86 | 1.56 ± 1.20 | 5.61 |

**^a^** average of the reference stations NS20 08, NS21 14 and VP21 48; **^b^** average of samples from above hydrothermal system VP20 05, NS21 09, TW21 38; **^c^** samples directly sampled from vents D20 01, D20 02, D21 03, D21 05, D21 07.

**Supplementary Figure S3.** Microbial Chao1 richness (A, B) and Shannon diversity (C, D) estimates for each survey. Note differences in y-axis scales. Note that 2020 survey named Colcarto-0220 and 2021 named Colcarto-0221.

**Supplementary Figure S4.** Relative abundance of prokaryotic 16S rRNA sequences classified by (A) family and (B) genus from all microbial mat samples. Only families present in more than two samples and belonging to classes with at least 1% median relative abundance are shown, with bold labels highlighting families exceeding 1% median relative abundance across all samples. Only genera with at least 0.1% median relative abundance that belong to classes with at least 1% median relative abundance are shown, ordered by decreasing abundance, and highlighted with bold labels when exceeding 1% median relative abundance across all samples. The color gradient indicates relative abundances ranging from <1% (yellow), 1-10% (orange), and 10-40% (red).

**Supplementary Figure S5.** A) Hierarchical clustering and relative abundance of class-classified eukaryotic 18S rRNA sequences (including Opisthokonta) collected during the 2020 (yellow) and 2021 (purple) surveys. The dendrogram includes approximately unbiased (au) values (grey) to indicate clustering uncertainty. Taxa are presented in decreasing abundance with classes equal to or below 1% median relative abundance grouped as “Other”. B) Relative abundance of families belonging to classes with median relative abundances higher than 1%. Boxplots show the median, first (upper) and third (lower) quartiles, and actual observations (points) for each survey. C) Relative abundances of genera belonging to the Maxillopoda family. The color gradient indicates relative abundance <1% (yellow), 1-10% (orange), and 10-50% (red). Note that 2020 survey named Colcarto-0220 and 2021 named Colcarto-0221.

**Supplementary Figure S6.** Relative abundance of eukaryotic 18S rRNA sequences (excluding Opisthokonta) classified by (A)family and (B) genus from all microbial mat samples. Only families present in more than two samples and belonging to classes with at least 1% median relative abundance are shown, with bold labels highlighting families exceeding 1% median relative abundance across all samples. Only genera with at least 0.1% median relative abundance that belong to classes with at least 1% median relative abundance are shown, ordered by decreasing abundance, and highlighted with bold labels when exceeding 1% median relative abundance across all samples. The color gradient indicates relative abundances ranging from <1% (yellow), 1-10% (orange), and 10-80% (red).

a’

a’

a

a

B

A

**Supplementary Figure S7.** Spatial distribution of fluorescence (A) and anomaly of fluorescence with respect to references (B) along TW21 38 (see Fig.1 and Fig. 3A) surveyed during 2021 cruise.

| **Cruise** | **Location** | **Id** | **Survey Method** | **Date** | **Ti** | **Tf** | **Lat i (N)** | **Lon i (E)** | **Lat f (N)** | **Lon f (E)** | **Total length (m)** | **Prof max (m)** |
| --- | --- | --- | --- | --- | --- | --- | --- | --- | --- | --- | --- | --- |
| Colcarto-0220 | Calent mound | NS 01 | Near-seabed tow | 11/2/20 | 12:04:11 | 15:22:09 | 39.83776 | 0.68106 | 39.83760 | 0.68280 | 2294.2 | 59.9 |
|  |  | NS 02 | Near-seabed tow | 12/2/20 | 12:01:19 | 15:10:48 | 39.83802 | 0.68050 | 39.83676 | 0.67984 | 2137.4 | 53.0 |
|  |  | TW 03 | Tow yo | 12/2/20 | 15:33:41 | 15:46:02 | 39.83546 | 0.68066 | 39.83546 | 0.68242 | 150.7 | 43.2 |
|  |  | VP 05 | Vertical Profile | 13/2/20 | 07:20:58 | 07:39:50 | 39.83528 | 0.68154 | 39.83528 | 0.68154 | 0 | 43.4 |
|  |  | NS 06 | Near-seabed tow | 13/2/20 | 09:35:57 | 13:08:01 | 39.84162 | 0.68040 | 39.83392 | 0.68336 | 2246.2 | 70.9 |
|  |  | NS 07 | Near-seabed tow | 14/2/20 | 10:19:46 | 12:24:05 | 39.83582 | 0.68340 | 39.83572 | 0.68516 | 1473.1 | 68.5 |
|  | Ref. stations | NS 08 | Near-seabed tow | 14/2/20 | 15:09:21 | 15:43:19 | 39.85904 | 0.71688 | 39.85840 | 0.71990 | 268.0 | 88.2 |
|  |  | NS 09 ref | Near-seabed tow | 14/2/20 | 16:33:19 | 17:00:00 | 39.83250 | 0.69236 | 39.83308 | 0.68990 | 220.2 | 95.4 |
|  |  | NS 10 | Near-seabed tow | 14/2/20 | 17:21:47 | 17:45:04 | 39.82214 | 0.69882 | 39.82288 | 0.69664 | 205.5 | 93.8 |
|  |  | NS 11 | Near-seabed tow | 14/2/20 | 18:20:40 | 19:32:48 | 39.83130 | 0.62396 | 39.83342 | 0.62280 | 766.3 | 85.3 |
|  |  | NS 12 | Near-seabed tow | 15/2/20 | 10:00:36 | 11:15:50 | 39.89476 | 0.63522 | 39.89476 | 0.63468 | 800.2 | 60.6 |
| Colcarto-0221 | Calent mound | NS 09 | Near-seabed tow | 14/2/21 | 18:21:10 | 18:45:49 | 39.83504 | 0.68312 | 39.83502 | 0.67944 | 573.2 | 54.6 |
|  |  | TW 38 | Tow yo | 19/2/21 | 18:10:04 | 19:03:33 | 39.83328 | 0.68280 | 39.83820 | 0.67942 | 856.8 | 51.0 |
|  | Ref. stations | NS 14 ref | Near-seabed tow | 15/2/21 | 18:18:53 | 18:49:24 | 39.82632 | 0.67972 | 39.83304 | 0.67450 | 1150.5 | 81.5 |
|  |  | TW 19 ref | Tow yo | 17/2/21 | 15:43:36 | 16:00:26 | 39.83702 | 0.68880 | 39.83726 | 0.68716 | 190.5 | 84.2 |
|  |  | TW 21 ref | Tow yo | 17/2/21 | 17:11:30 | 18:44:26 | 39.82692 | 0.68140 | 39.83486 | 0.67610 | 1554.2 | 85.0 |

**Supplementary Table S8.** Sampling information for the oceanographic study carried out in 2020 (Colcarto-0220) and 2021 (Colcarto-0221).

**Supplementary Table S9.** Sampling metadata and number of sequences reads in each library (before and after rarefaction) of microbial mat samples collected during Colcarto1-0220 (year 2020) and Colcarto-0221 (year 2021) surveys.

| **Sample ID** | **Sampling** | | | | | **16S rRNA** | | **18S rRNA** | |
| --- | --- | --- | --- | --- | --- | --- | --- | --- | --- |
|  | **Cruise** | **Latitude (ºN)** | **Longitude (ºE)** | **Depth (m)** | **Visual aspect** | **Total reads** | **Rarefied reads** | **Total reads** | **Rarefied reads** |
| S25 | Colcarto-0220 | 39.83512 | 0.68208 | 40 | Yellow mat coloration | 71,119 | 36,709 | 29,785 | 27,358 |
| S26 | Colcarto-0220 | 39.83512 | 0.68208 | 40 | Red mat coloration | 36,709 | 36,709 | 27,358 | 27,358 |
| S27 | Colcarto-0220 | 39.83512 | 0.68208 | 40 | White mat coloration | 81,577 | 36,709 | 39,566 | 27,358 |
| S28 | Colcarto-0221 | 39.83512 | 0.68208 | 44 | - | 62,142 | 36,709 | 30,285 | 27,358 |
| S29 | Colcarto-0221 | 39.83512 | 0.68208 | 44 | - | 89,268 | 36,709 | 34,118 | 27,358 |
| S31 | Colcarto-0221 | 39.83512 | 0.68208 | 44 | - | - | - | 53,446 | 27,358 |
